# Supplementary material for: The Position and Complex Genomic Architecture of Plant T-DNA Insertions Revealed by 4SEE
Source: Int J Mol Sci. 2020 Mar 30;21(7):2373. doi: 10.3390/ijms21072373 (PMC7177604; doi:10.3390/ijms21072373)

Supplemental Figure S1- Endogenous 4C *cis* signal.

4C view of **(A)** *SBT4.12 (*At5G59090), **(B)** *PIF4 (*At2G43010) and (**C)** *MBP1 (*AT1G52040). In each panel, 4C-seq profile of the entire *cis* chromosome is indicated in gray above the local peak. Centromeres are marked as blue rhombus and the genic position of the viewpoint and the main 4c signal is marked in a red rectangle. Below, browser shot zooming in to the viewpoint region is shown. Genomic position of the viewpoint is indicated with a dashed red line.

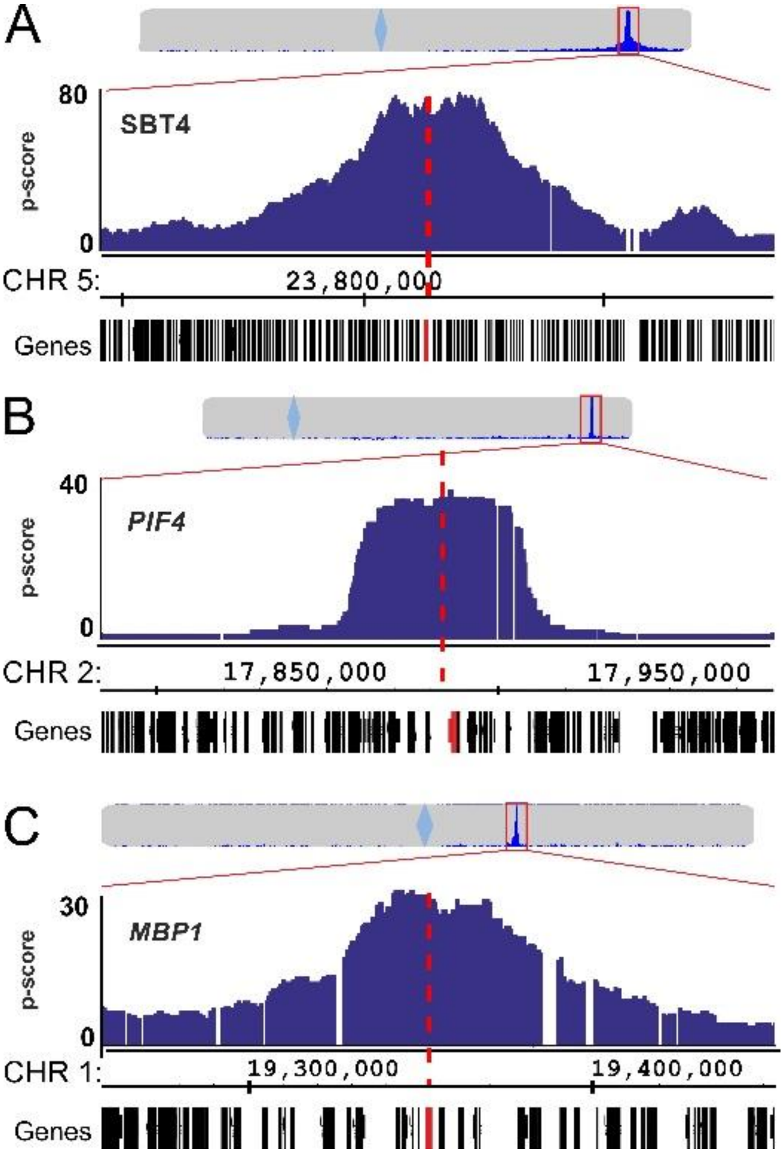

Supplemental Figure S2. T-DNA insertion validated by deep sequencing of PE reads from ADF8p:NTF/ACT2p:BirA transgene.

Number of PE reads that were mapped to the T-DNA plasmid and the genome (out of 69,166,811) from GSE101482. Loci were retrieved also by TDNAscan: Chr1:194,132, Chr4:15,978,987, Chr5:9,939,147, and Chr5:26,902,554, are shaded in gray.

Locus	Number of reads	
Chr1:193817-194253	31	ADF8 T-DNA
Chr4:280384-281374	28	Endogenous ADF8
Chr4:15978419-15978783	13	ADF8 T-DNA
Chr4:18149292-18149872	83	BirA T-DNA
Chr5:9938731-9938961	23	ADF8 T-DNA
Chr5:26902115-26902340	34	ADF8 T-DNA
Chr1:24511209-24511214	2	
Chr2:2209792-2209798	2	
Chr3:2094491-2094493	2	

Number of PE reads that were mapped to the T-DNA plasmid and the genome (out of 21,138,263) from GSE53324. Out of these loci TDNAscan retrieved only the endogenous *ADF8* locus.

Locus	Number of reads	
Chr1:193682-193883	5	ADF8 T-DNA
Chr4:280782-281117	12	Endogenous ADF8
Chr4:15978569-15978778	6	ADF8 T-DNA
Chr4:18149463-18149620	6	BirA T-DNA
Chr5:9938912-9938964	3	ADF8 T-DNA
Chr5:26902181-26902319	4	ADF8 T-DNA

Supplemental Figure S3. Fluorescent in situ hybridization (FISH) on somatic nuclei from *Arabidopsis thaliana* WT (**A-C**) and ADF8p:NTF/ACT2p:BirA transgenic plants (**D-F**). F1H21 probe on chromosome 5 was labeled in green, 10-O-11 probe on chromosome 1 was labeled in red, DNA counterstaining with DAPI in blue.

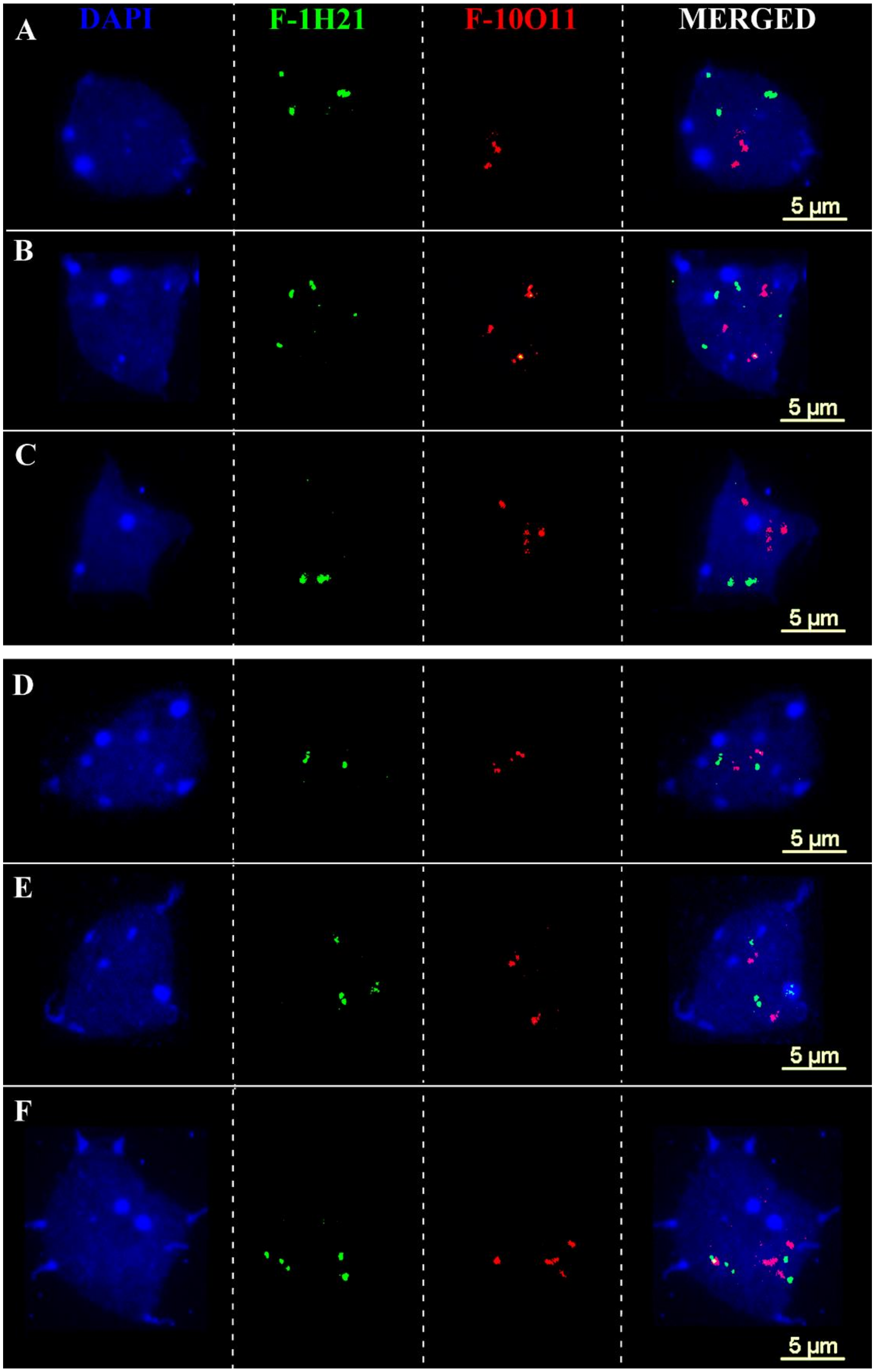

Supplemental Figure S4. Mapping chromosomal rearrangements in ADF8p:NTF/ACT2p:BirA line.

Browser shots of 4C profiles demarcating the chromosomal breakpoints and fusions (marked with a dashed red line) in ADF8p:NTF/ACT2p:BirA line. **(A)** Shows the excision of fragment 5.2 and fusion between 5.1 and 5.3. The continuous profile of *BON2* across 5.2 and 5.1 in WT plants (second row) is broken at the transition point in the transgenic line (first row). The continuous profiles of *Myblike* and *PP2Cf* across 5.2 and 5.1 fragments in WT plants (second and 6th rows) is broken in the transgenic line (first and 5th rows) at the borders with the 5.2 fragment, indicating the discontinuous DNA molecule. **(B)** Shows the 4C signal of *SBT4.12* (located on fragment 5.3) on fragment 1.1 in ADF8p:NTF/ACT2p:BirA (second row) as indication for the fusion between 5.3 and 1.1. The continuous profile of *EMB76* across 1.1 and 1.2 in WT plants (4th row) is broken at the transition point in the transgenic line (5th row), indicating the discontinuity. **(C)** High 4C signal of *BON2* (located on fragment 5.2) on fragment 5.4 indicates the fusion between the two fragments in ADF8p:NTF/ACT2p:BirA (second row). *SBT4.12* and RT5 profiles demarcate the break between 5.3 and 5.4. *ADF8* profile (5th row) indicates the location of the T-DNA at the border of fragment 5.3. Arrows at the top mark the viewpoint position, Y axis demarcated read count, chromosomal coordinates of *Arabidopsis thaliana* TAIR10 genome build are indicated.

A

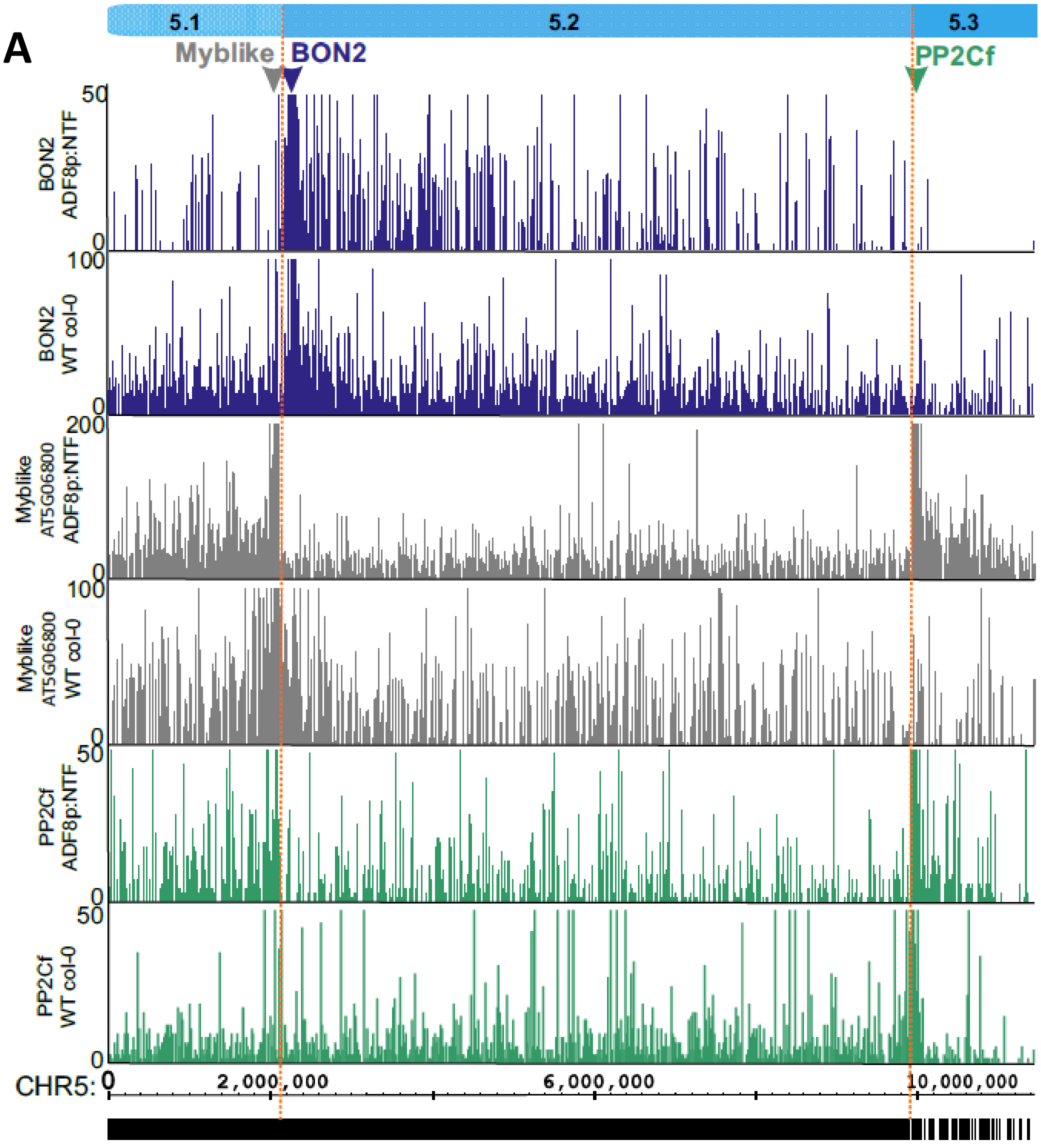
5.1
5.2
5.3
Myblike
BON2
PP2Cf
BON2 ADF8p:NTF
BON2 WT col-0
Myblike AT5G06800 ADF8p:NTF
Myblike AT5G06800 WT col-0
PP2Cf ADF8p:NTF
PP2Cf WT col-0
50
0
100
200
CHR5:
0
2,000,000
6,000,000
10,000,000

B

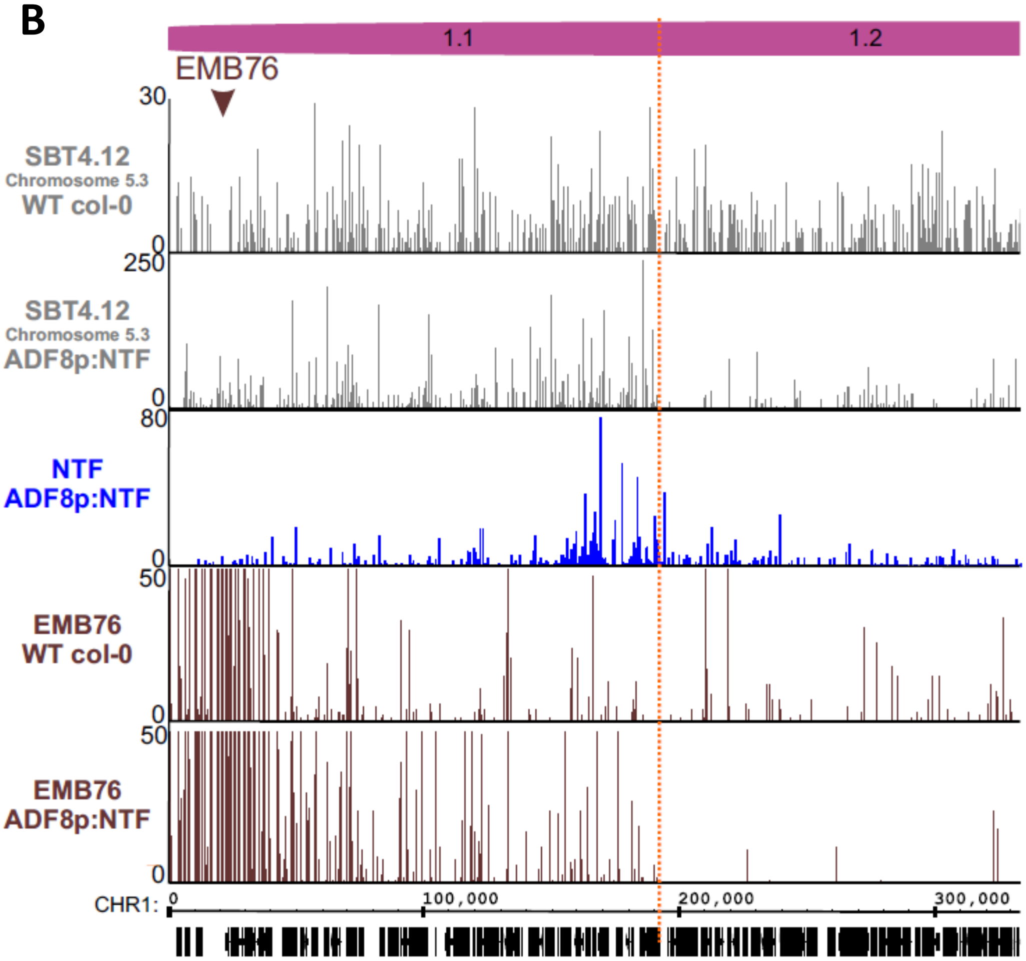

1.1
1.2
EMB76
30
SBT4.12
Chromosome 5.3
WT col-0
0
250
SBT4.12
Chromosome 5.3
ADF8p:NTF
0
80
NTF
ADF8p:NTF
0
50
EMB76
WT col-0
0
50
EMB76
ADF8p:NTF
0
CHR1:
0
100,000
200,000
300,000

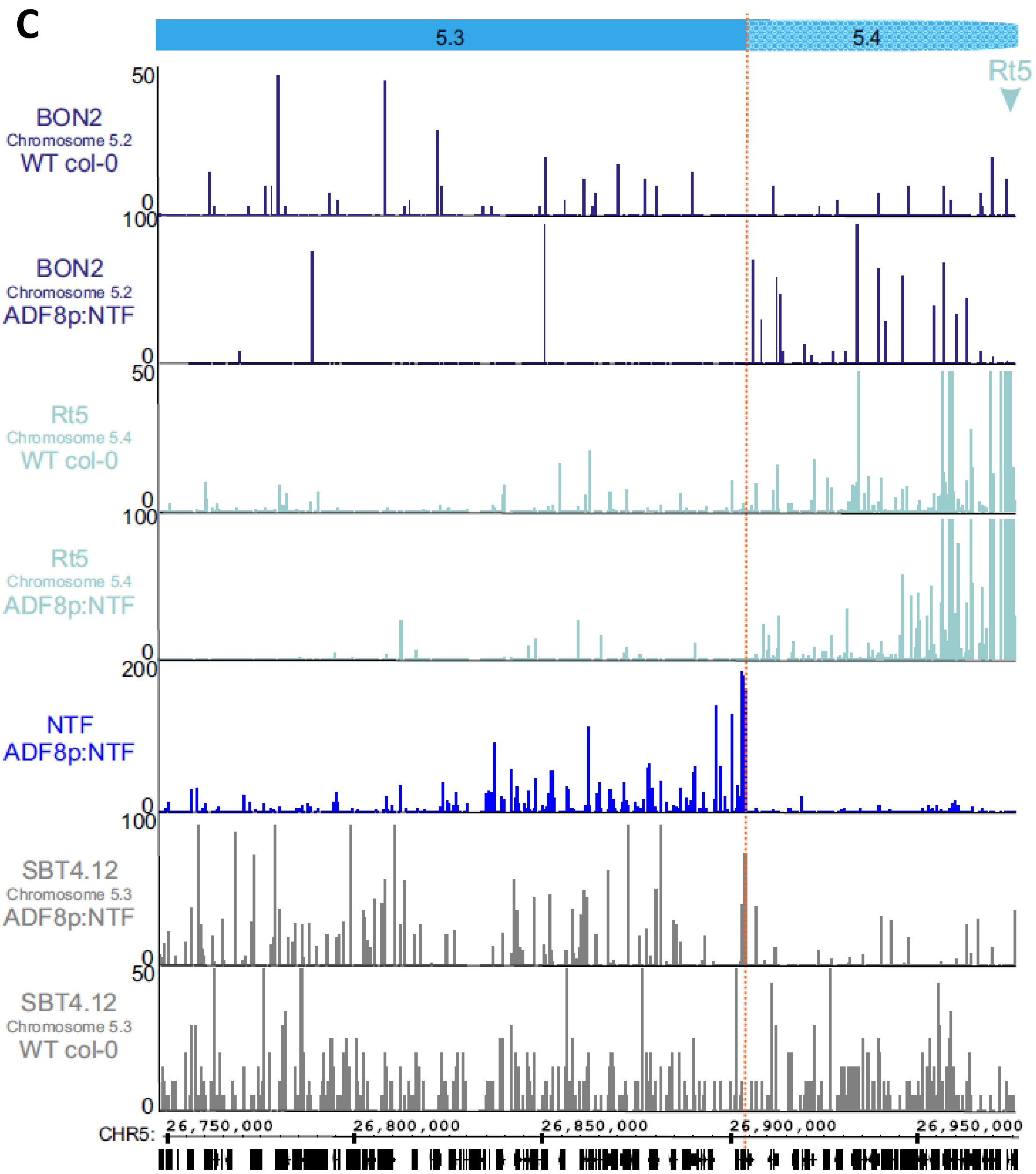
C
5.3
5.4
Rt5
BON2
Chromosome 5.2
WT col-0
BON2
Chromosome 5.2
ADF8p:NTF
Rt5
Chromosome 5.4
WT col-0
Rt5
Chromosome 5.4
ADF8p:NTF
NTF
ADF8p:NTF
SBT4.12
Chromosome 5.3
ADF8p:NTF
SBT4.12
Chromosome 5.3
WT col-0
CHR5:
26,750,000
26,800,000
26,850,000
26,900,000
26,950,000

Supplemental Figure S5. Chromosomal translocations on chromosome 5 validated by PE sequencing ATAC-seq data. (A) Browser view of the fusion between 5.1 (shown Chr5:2103500-2113500) and 5.3 (shown Chr5: 9938000-9947999). The fusion point is marked in a dashed line. Alignment of paired end reads from ADF8p:NTF/ACT2p:BirA DHS-seq (GSE53324) and ATAC-seq (GSE101482) experiments are displayed in the two top panels. Alignment of ATAC-seq (GSE101482) from Gl2:NTF/ACT2p:BirA plants, serving as a negative control is at the bottom panel. Reads are displayed as pairs. Scale bar relative to the dashed line is presented at the bottom. The genomic location of the fusion is indicted on the top. (**B)** same as A, for the fusion of fragments 5.2 (shown Chr5:26902300-26902700) and 5.4 (shown Chr5:2113600-2113800).

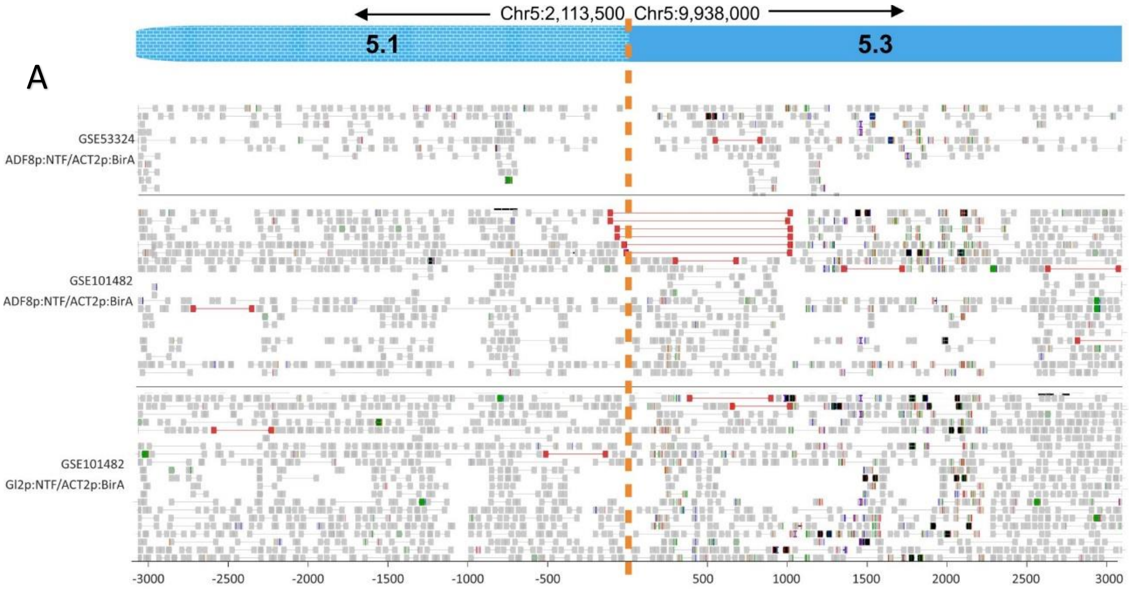

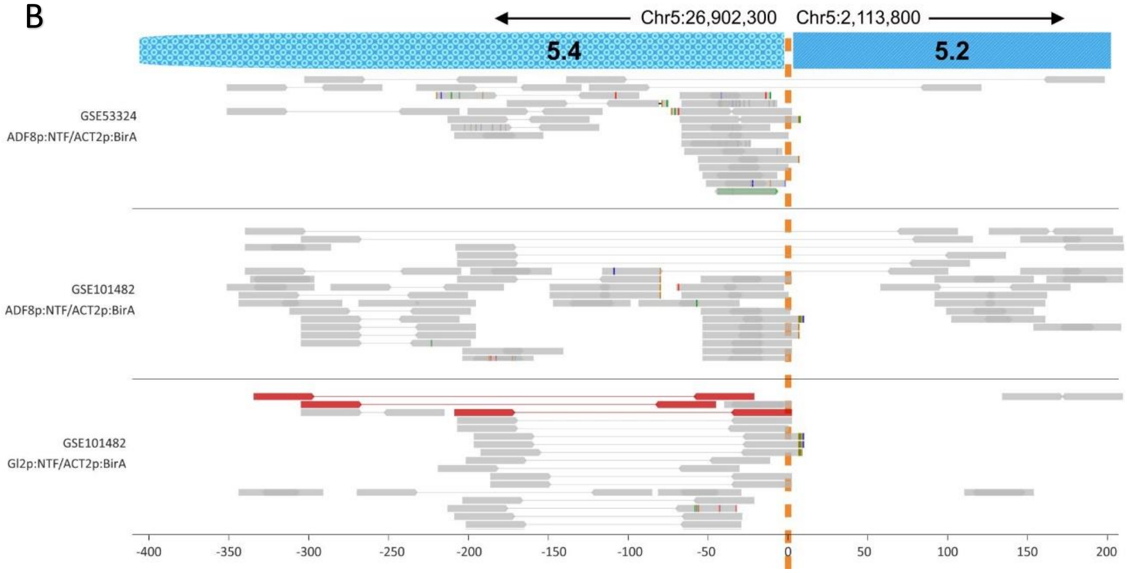

Supplemental Figure S6. Genomic coverage of 4C-seq for SALK_063720 and SALK_064627 in different template amounts. The prominent peaks on chromosome 2 in SALK_063720, and on chromosomes 1 and 3 in SALK_064627 demarcate the T-DNA insert.

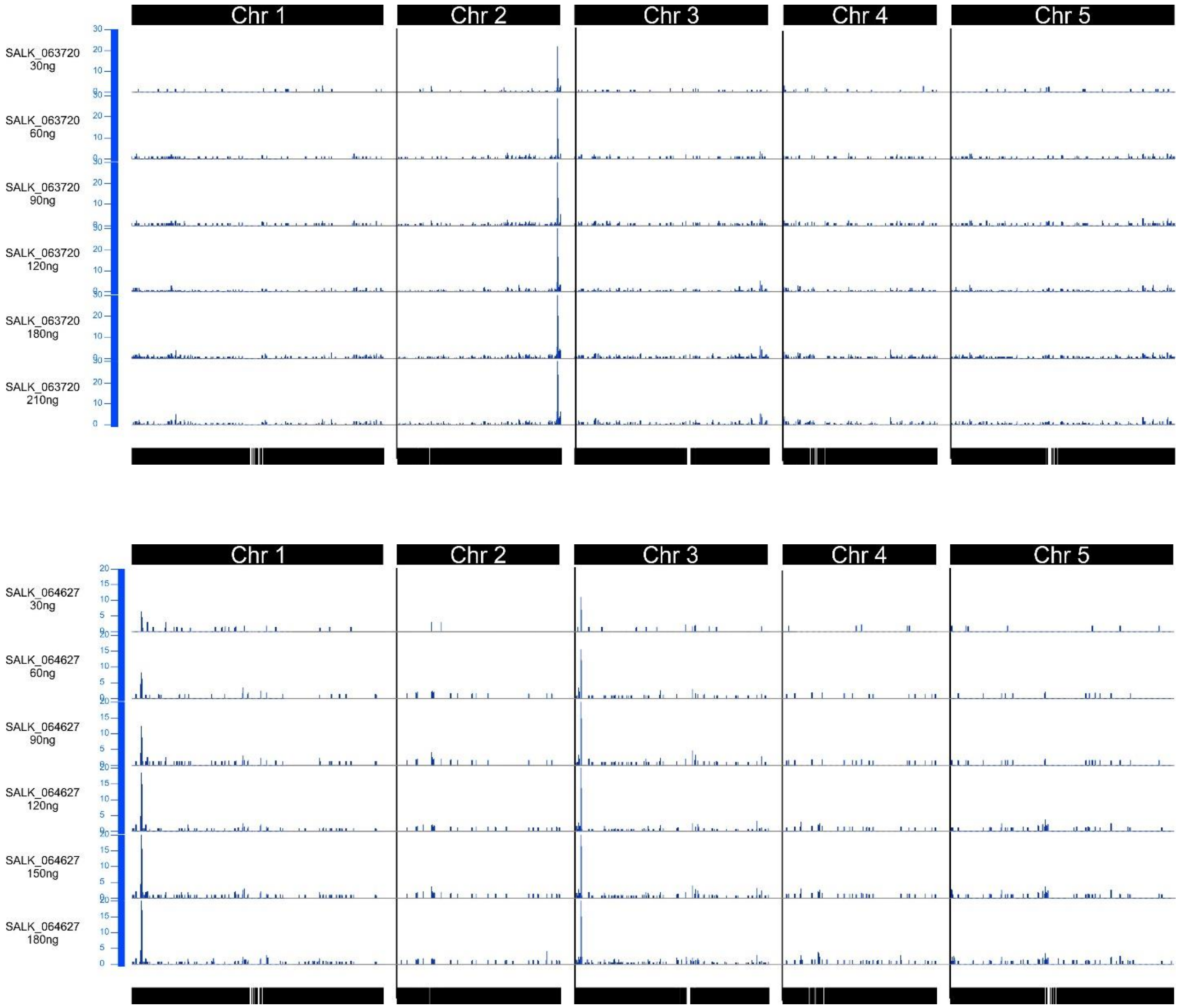

Supplemental Figure S7. Genomic distribution of peak centers at 50% cutoff in SALK lines. Position of the insertion site is marked in red, median of the positions of the peak centers is marked in green and their average in blue. Peak centers position and the specific experiment (ng of input DNA) are indicated above and the genomic coordinates below the horizontal axis.

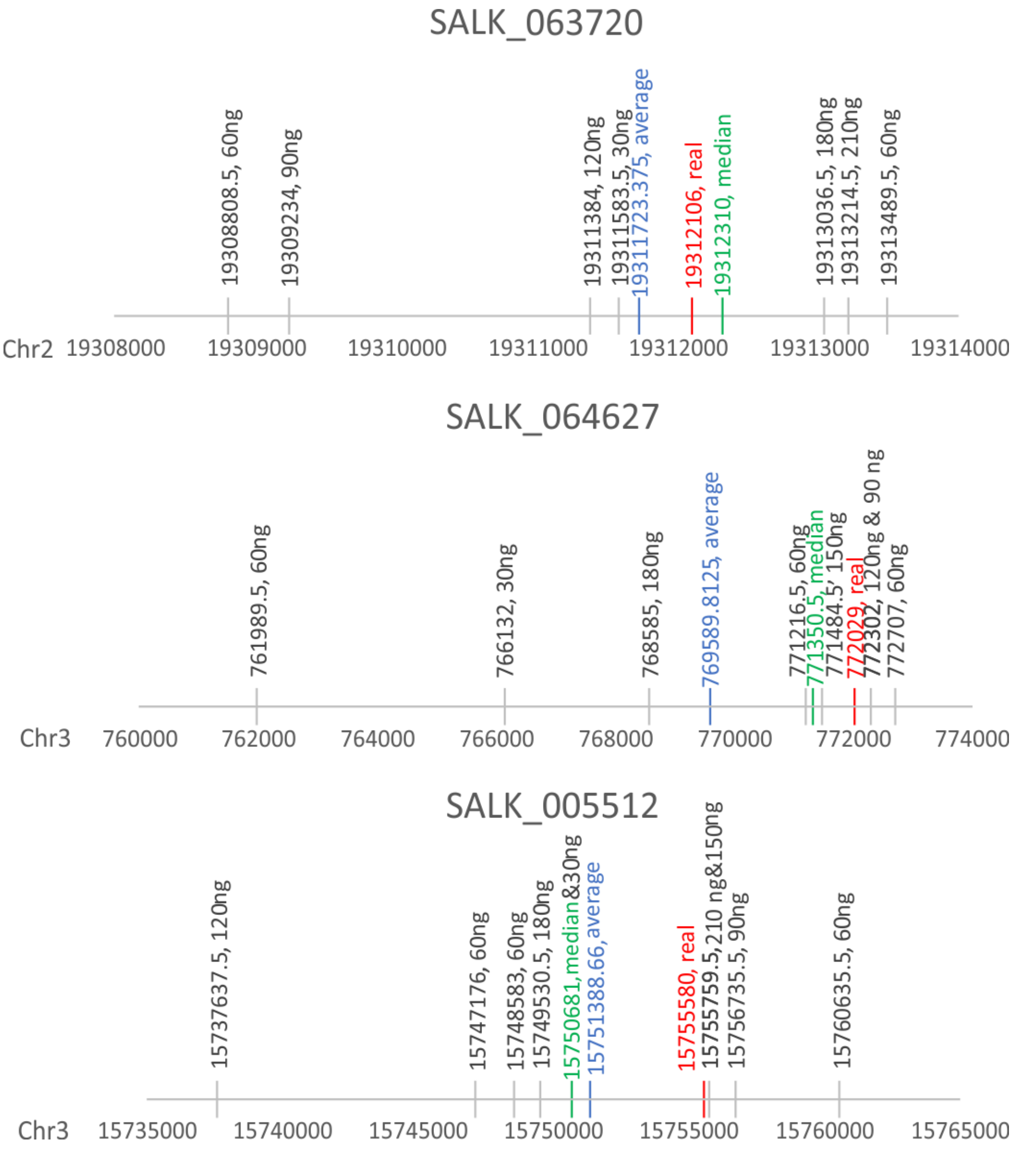

Supplement: Supplementary file 1 [file ijms-21-02373-s001.zip › sup_figs_proofs.pdf]
